# Exploration of the Molecular Mechanism by Which Caveolin-1 Regulates Changes in Blood–Brain Barrier Permeability Leading to Eosinophilic Meningoencephalitis

**DOI:** 10.3390/tropicalmed9060124

**Published:** 2024-05-24

**Authors:** An-Chih Chen, Shih-Chan Lai, Cheng-You Lu, Ke-Min Chen

**Affiliations:** 1 Department of Neurology, Chung Shan Medical University Hospital, Taichung 40201, Taiwan; cshy1135@csh.org.tw; 2 Department of Parasitology, Chung Shan Medical University, Taichung 40201, Taiwan; shih@csmu.edu.tw; 3 Clinical Laboratory, Chung Shan Medical University Hospital, Taichung 40201, Taiwan; 4 Department of Post-Baccalaureate Medicine, College of Medicine, National Chung Hsing University, Taichung 40227, Taiwan; cylu150@dragon.nchu.edu.tw

**Keywords:** *Angiostrongylus cantonensis*, Caveolin-1, blood–brain barrier permeability, cerebral edema

## Abstract

*Angiostrongylus cantonensis*, a zoonotic parasite, can invade the human central nervous system (CNS) and cause acute eosinophilic meningitis or eosinophilic meningoencephalitis. Mice infected with *A. cantonensis* show elevated levels of pro-inflammatory cytokines, plasminogen activators, and matrix metalloproteinase-9, resulting in disruption of the blood–brain barrier (BBB) and immune cell infiltration into the CNS. Caveolin-1 (Cav-1) regulates the permeability of the BBB, which affects immune cells and cerebrospinal fluid. This intricate interaction ultimately fuels the progression of brain damage and edema. This study aims to investigate the regulatory role of Cav-1 in the pathogenesis of meningoencephalitis induced by *A. cantonensis* infection. We investigated pathological alterations by triphenyl-tetrazolium chloride, brain water content, BBB permeability, Western blot analysis, and gelatin zymography in BALB/c mice after *A. cantonensis*. The study evaluates the critical role of Cav-1 regulation through the TLR4/MyD88 signaling pathway, modulates tight junction proteins, influences BBB permeability, and contributes to brain damage in *A. cantonensis*-induced meningoencephalitis.

## 1. Introduction

*Angiostrongylus cantonensis* is a kind of zoonotic parasite [1], *A. cantonensis* larvae can invade the human central nervous system and cause acute eosinophilic meningitis or eosinophilic meningoencephalitis, and in the most severe infection, increased brain pressure, increased cerebrospinal fluid pressure, coma, and death [2]. Pathological mechanisms causing brain damage include physical destruction of tissues by larval migration [3], and immune responses induced by *A. cantonensis* infection include the hippocampus and brain. Within the parenchyma, astrocytes, microglia, and neurons undergo apoptosis, necroptosis, or demyelination. Additionally, there is a loss of Purkinje cells in the cerebellum, disruption of the blood–brain barrier (BBB), and various other pathological phenomena [4,5,6,7,8,9].

The neurovascular unit is made up of cells including endothelial cells, pericytes and vascular smooth muscle cells, and glial cells. Stable and proper interaction among all components of the neurovascular unit is essential for the stability and function of the central nervous system. It is inevitable that if one of the members is out of balance, it will cause the appearance of the disease [10]. Caveolin (Cav) and cholesterol collaborate to shape and maintain the architecture of caveolae. Cav-1 adopts a hairpin-shaped configuration within the cellular membrane, with the N- and C-terminals extending into the cytoplasm. These terminals facilitate binding to various receptors and numerous message-transmitting molecules through the caveolin scaffold domain. However, the impact of these interactions on downstream cell signaling pathways, whether positive or negative, depends on the specific cell type, stimuli, and induced cellular signaling pathways [11,12,13,14]. Cav-1 participates in the mechanism of regulation of physiology and pathology through a complex information transmission pathway system, such as endocytosis, cholesterol regulation of transport, lipid metabolism, transcellular trafficking, and immune response [15,16,17]. Cav-1 is present in endothelial cells, astrocytes, and neurons in the neurovascular unit of the brain, and is especially abundant in endothelial cells [18,19,20,21]. Cav-1 changes BBB permeability of the BBB by regulating the endocytosis of vascular endothelial cells and intercellular tight junction proteins and adherent junction proteins. The degradation of the extracellular matrix in the basal layer, transmembrane proteins, and tight junction proteins between endothelial cells will lead to the destruction of the BBB structure, resulting in the leakage of immune cells, red blood cells, and fluid from the blood vessels to the central nervous system [10,15,16,17,22]. Cav-1 participates in the physiological and pathological mechanisms of many brain lesions and brain edema processes, including the regulation of immune responses and BBB communication. Therefore, the role of Cav-1 in the regulation of BBB damage and permeability after brain injury remains controversial.

In mice infected with *A. cantonensis*, it can cause eosinophilic meningitis, brain injury, cerebral hemorrhage, and cerebral edema. There are still many mechanism that remain unclear. This study clarifies that Cav-1 plays a role in the promotion of inflammation and damage to the BBB in hemorrhagic brain injury caused by *A. cantonensis*-induced eosinophilic meningoencephalitis and cerebral edema, increasing the permeability of the BBB or inhibiting the immune response to protect the brain and reduce damage.

## 2. Materials and Methods

### 2.1. Collection of Third-Stage Larvae (AcL3) of A. cantonensis

After infecting rats with *A. cantonensis*, their feces contained first-stage larvae (AcL1) of *A. cantonensis*. These AcL1 larvae were collected from feces to infect water snails (Biomphalaria glabrata). After 60 days of infection, the AcL1 larvae matured into third-stage larvae (AcL3). The tissue of the water snails was homogenized in a ratio of 1:30 (tissue: artificial pepsin digestion solution containing pepsin from Sigma, St. Louis, MO, USA) using a digestive juice grinder. The mixture was then evenly stirred and digested in a 37 °C incubator for 1 h. The lower layer of sediment was carefully collected using a dropper, transferred to a glass dish, and observed under a dissecting microscope to identify and collect the AcL3. AcL3 were then quantified, and every 30 larvae were grouped into a 1.5 mL microcentrifuge tube for future feeding experiments with mice.

### 2.2. Induction of Animal Models of Eosinophilic Meningitis

Sixty-three BALB/c mice were randomly assigned into seven groups, each consisting of nine mice. These groups included the uninfected control group, the group infected with *A. cantonensis*, and the group infected with *A. cantonensis* and treated with Methyl-β-cyclodextrin (MβCD). In the groups infected with *A. cantonensis* and treated with MβCD, each mouse received 30 AcL3. Mice in the *A. cantonensis* infection group were sacrificed on days 5, 10, 15, 20, and 25 after infection for time course experiments. The non-infected control group received an equivalent volume of deionized water on day 0, as did the infected groups, and were euthanized on day 25. In the MβCD treatment group, MβCD (Merck, Rahway, NJ, USA) was administered intraperitoneally at a dose of 100 mg/kg/day from day 0 of *A. cantonensis* infection to day 24, with euthanasia of mice on day 25 after infection.

### 2.3. Cerebral Edema Determination

Mice were anesthetized with isoflurane and sacrificed by decapitation. The brains were removed and washed with phosphate buffered saline (PBS). The appearance of the brain was monitored for hemorrhage and infection by *A. cantonensis*, and then the brain tissue was placed in cold PBS for 10 min and then placed in a brain tissue slice mold to cut into brain slices with a thickness of 1 mm, infiltrated with 2% TTC (2, 3, 5-triphenyl-tetrazolium chloride solution) at 37 °C and kept away from staining. This was carried out for 30 min in a light environment and then the brain tissue was fixed with 10% formaldehyde for 24 h. After scanning, the volume of the brain was calculated with an image processing system (AIS software, version 4.0, Imaging research Inc., Catharines, ON, Canada).

### 2.4. Measurement of Brain Water Content

The water content of the brain was measured using the wet dry weight method, and then the mice were sacrificed to remove brain tissue and weighed as wet weight. Brain tissue was fully dried in a constant temperature oven at 80 °C for 24 h and weighed several times to obtain a stable dry weight (average weighing error < 0.002 g). The percentage of brain water content was calculated as follows: brain water content = (wet weight − dry weight)/wet weight × 100%.

### 2.5. Measurement of BBB Permeability

The permeability of the BBB was examined by an in vivo permeability assay. To this end, 40 kDa FITC-dextran (6 mg/mL, 4 mg/kg body weight, Sigma, St. Louis, MO, USA) was injected into the tail vein. The control group was injected with normal saline. After 30 min, the mice were anesthetized with isoflurane and CSF was sucked through the dura mater with a capillary tube. Excitation: 490 and emission: 520 nm were used to measure absorbance on a spectrophotometer (Hitachi U4500, Tokyo, Japan) and the 40 kDa FITC-dextran concentration in CSF was calculated using the standard curve.

### 2.6. Western Blot

The protein detection method followed the procedures described in previous research conducted in our laboratory [8]. In summary, the sample was centrifuged at 12,000× *g* for 10 min at 4 °C and the resulting supernatant was collected and quantified. The freshly extracted proteins were then loaded onto the SDS electrophoresis page for electrophoresis. After electrophoresis, the proteins on the page were transferred onto a PVDF membrane. A primary antibody was used followed by a horseradish peroxidase (HRP) conjugated secondary antibody for the reaction, which facilitates the detection of proteins through enhanced chemiluminescence (ECL). Subsequently, the protein bands were analyzed using a cold-light imager (iBright CL750 imaging system, Thermo, Waltham, MA, USA).

### 2.7. Gelatin Zymography

Brain tissue protein was loaded onto 7.5% (mass/volume) SDS-polyacrylamide gel electrophoresis after copolymerization with 0.1% gelatin (Sigma, St. Louis, MO, USA). The stacking gels were 4% (mass/volume) polyacrylamide. Electrophoresis was carried out in running buffer (25 mM Tris, 250 mM glycine, 1% SDS) at room temperature at 110 V for 1 h. The gel was washed twice at room temperature for 30 min each in 2.5% Triton X-100 and then twice with double-distilled H_2_O for 10 min each. The gel was incubated in reaction buffer (50 mM Tris-HCl, pH 7.5, containing 200 mM NaCl, 10 mM CaCl_2_, 0.02% Brij-35, 0.01% NaN_3_) at 37 °C for 18 h. The gel was stained with 0.25% Coomassie Brilliant Blue R-250 (Sigma, St. Louis, MO, USA) for 1 h and destained in 15% methanol/7.5% acetic acid.

### 2.8. Statistical Analysis

The test results obtained from the different groups were statistically analyzed by Kruskal–Wallis, and multiple comparisons were made by Dunn’s multiple comparison. The results are expressed as mean ± standard deviation (means ± SD), and *p* < 0.05 means statistically significant.

## 3. Results

### 3.1. Alterations in the Permeability of the BBB Result in Cerebral Edema and Hemorrhage

**Figure 1:**
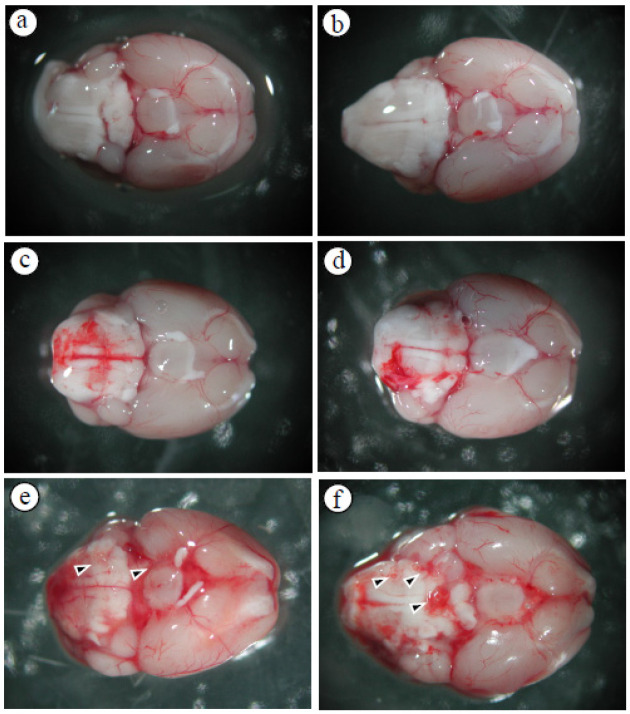
Cranial base hemorrhage in mice infected with *A. cantonensis* (**a**) control, (**b**) day 5, (**c**) day 10, (**d**) day 15, (**e**) day 20, (**f**) day 25. After infecting mice with *A. cantonensis*, the cranial base of the brain began to have a slight bleeding on day 10, and bleeding and expansion and congestion became more obvious on days 15 to 25, and the fifth stage of *A. cantonensis* could be found on days 20 and 25 (arrows).

**Figure 2:**
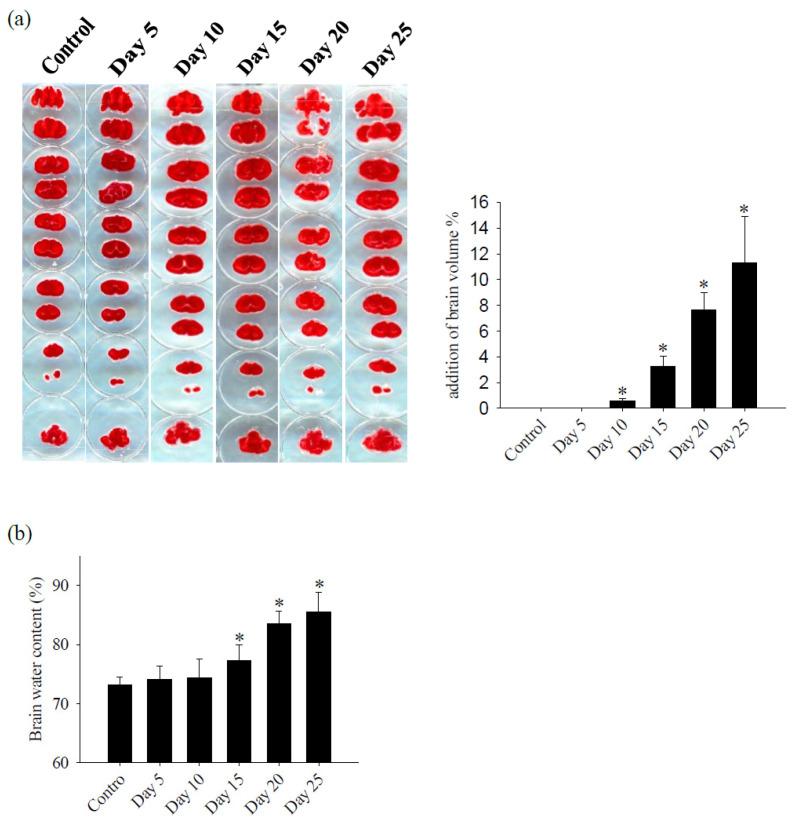
Changes in brain volume and edema. (**a**) Brain slices were stained with TTC, the volume of the brain was calculated by the image processing system (AIS software version 4.0, Imaging research INC, Canada) and (V_Day_ − V_control_)/V_control_ × 100% was used to calculate the percentage of increase in cerebellar volume after infection with *A. cantonensis*, the area was found to increase and edema on days 10 to 25 after infection with *A. cantonensis*. (**b**) Measured by water content, cerebral edema increased significantly on days 15–25 after infection with *A. cantonensis*. Data for three mice in each group are expressed as Mean ± SD, and * indicates a significant difference compared to the control group (*n* = 3, *p* < 0.05).

**Figure 3:**
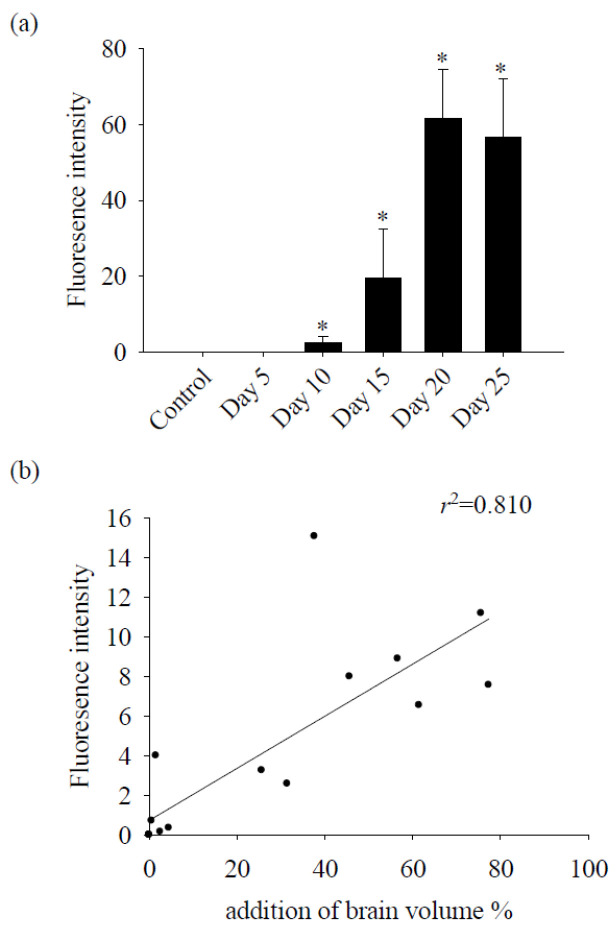
Correlation analysis between 40 kDa FITC-dextran fluorescence intensity and brain edema. (**a**) The fluorescence intensity of 40 kDa FITC-dextran in the CSF of mice infected with *A. cantonensis* on days 10 to 25 increased significantly. Data for three mice in each group are expressed as Mean ± SD, and * indicates a significant difference compared to the control group (*n* = 3, *p* < 0.05). (**b**) The fluorescence intensity of 40 kDa FITC-dextran in the CSF and brain edema after mice were infected with *A. cantonensis* (*n* = 3, *r*^2^ = 0.810, *p* < 0.05).

Following *A. cantonensis* infection in mice, a progressive increase in brain hemorrhage was consistently observed, as well as swelling and congestion of blood vessels throughout infection. In particular, five-stage larvae of *A. cantonensis* were detected at the base of the skull on days 20–25 after infection (Figure 1). Subsequent evaluation of brain volume revealed a significant increase on day 10 and showed a positive correlation with the duration of infection (Figure 2a). In addition, TTC staining revealed a 12% increase in brain volume and an 8% increase in brain water content on day 25 after infection (Figure 2b). Furthermore, the fluorescence intensity of 40 kDa FITC-dextran was examined in CSF and showed a notable increase, commencing on day 10 after infection (Figure 3a). Subsequent correlation analysis between this intensity and brain volume revealed a significant positive correlation (*r*^2^ = 0.810, *p* < 0.05) (Figure 3b). However, brain water content did not significantly increase until day 15 post-infection. These findings suggest that *A. cantonensis* infection induces alterations in BBB permeability, leading to changes in the permeability and, subsequently, causing cerebral edema and hemorrhage.

### 3.2. Cav-1 through the TLR4/MyD88 Signaling Pathway Modifies BBB Permeability

**Figure 4:**
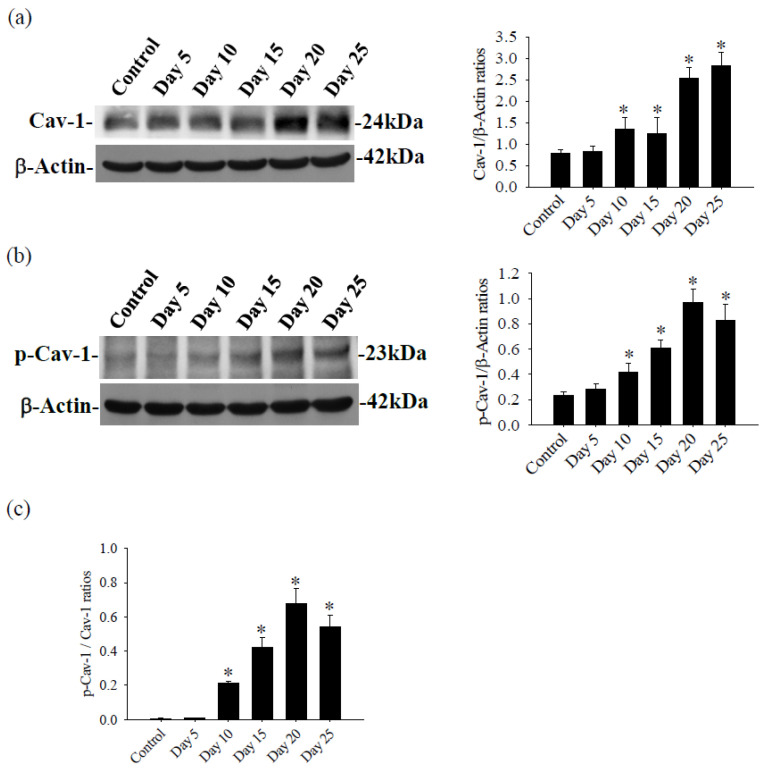
Expression of the Cav-1 and p-Cav-1 proteins in the brains of mice infected with *A. cantonensis*. (**a**) Western blot detection of p-Cav-1 protein expression, quantification of p-Cav-1 expression bands by the iBright CL750 imaging system increases significantly on days 10 to 25 after infection. (**b**) Western blot detection of p-Cav-1 protein expression, the quantification of p-Cav-1 expression by the iBright CL750 imaging system increases significantly on days 10 to 25 after infection. β-Actin is the loading control. (**c**) The Cav-1/p-Cav-1 ratios increases significantly on days 10 to 25 after infection. Data for three mice in each group are expressed as Mean ± SD, and * indicates a significant difference compared to the control group (*n* = 3, *p* < 0.05).

**Figure 5:**
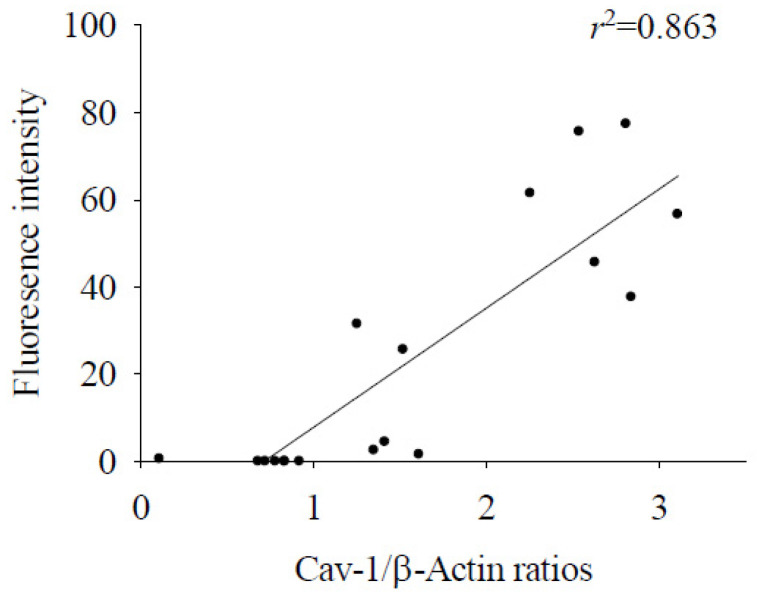
Correlation analysis between 40 kDa FITC dextran fluorescence intensity and Cav-1 protein. The fluorescence intensity of 40 kDa FITC dextran in the CSF showed a significant correlation with the expression of the Cav-1 protein in the brain (*n* = 3, *r*^2^ = 0.863, *p* < 0.05).

**Figure 6:**
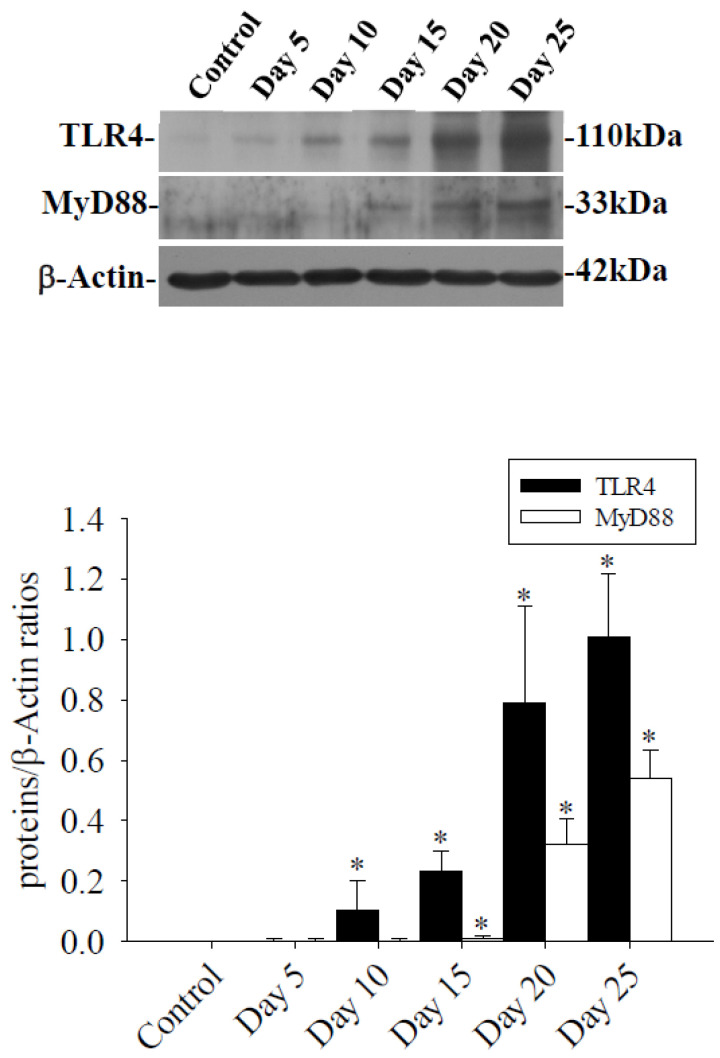
Expression of TLR4 and MyD88 proteins in the brains of mice infected with *A. cantonensis*. Western blot to detect the expression of the TLR4 and MyD88 protein, the band quantified by the iBright CL750 imaging system, the expression of TLR4 increased significantly on days 10 to 25 after infection, and the expression of MyD88 expression increased significantly on days 15 to 25 after infection. β-Actin is the loading control. Data for three mice in each group are expressed as Mean ± SD, and * indicates a significant difference compared to the control group (*n* = 3, *p* < 0.05).

In the brains of mice infected with *A. cantonensis*, there was a significant increase in Cav-1 levels, p-Cav-1 levels, and p-Cav-1/Cav-1 ratios on day 10 after infection, with a subsequent increase observed as infection progressed (Figure 4). The analysis correlating of 40 kDa FITC-dextran with the Cav-1 protein revealed a positive association (*r*^2^ = 0.863, *p* < 0.05, Figure 5). Furthermore, previous findings indicated a positive correlation between the fluorescence intensity of 40 kDa FITC-dextran in the CSF and the volume of the brain. Investigation of the Cav-1 regulatory pathway of Cav-1 revealed a significant elevation in the TLR4 and MyD88 signaling pathways in the brains of mice infected with *A. cantonensis* on days 10 and 15 after infection (Figure 6), which is consistent in agreement with the observed increase in the Cav-1 and p-Cav-1 proteins during the same time frame. This study proposes that Cav-1 regulates BBB permeability through the TLR4/MyD88 signaling pathway.

### 3.3. Treatment with Caveolae/Cav-1 Specific Inhibitor MβCD

**Figure 7:**
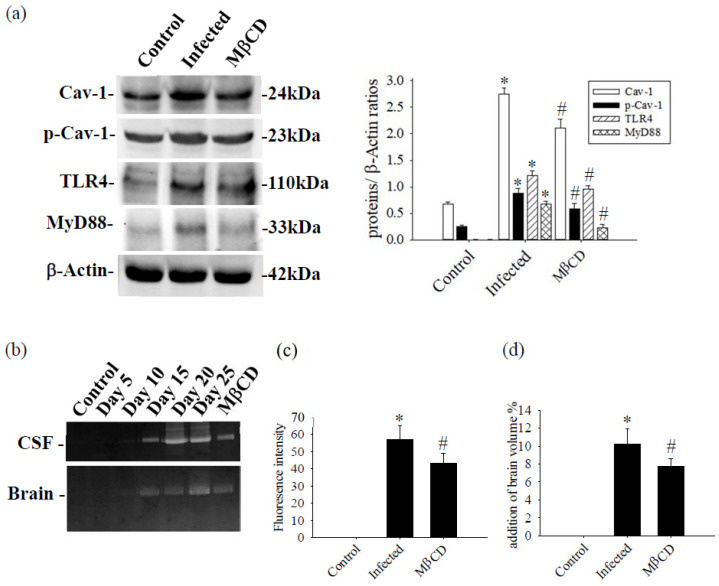
The Caveolae/Cav-1 specific inhibitor MβCD was treated with mice infected with *A. cantonensis*. (**a**) The levels of Cav-1, p-Cav-1, TLR4, and MyD88 proteins exhibited significant increases in the infection group compared to the control group, but significantly decreased in the MβCD treatment group compared to the infection group. β-Actin is the loading control. (**b**) In the brain and CSF of MMP-9 activation measured by zymography, MMP-9 activity increased significantly on day 15 and reached a peak on days 15 and 20. MMP-9 activity decreased significantly in the group treated with MβCD treatment. (**c**) The 40 kDa FITC-dextran fluorescence intensity in CSF increased significantly in the infected group and decreased significantly in the group treated with MβCD treatment. (**d**) Changes in brain volume observed with TTC stain. Brain volume increased significantly and decreased significantly in the group treated with MβCD treatment. Data from three mice in each group are expressed as Mean ± SD, * indicates a significant difference compared to the infected group with the control group (*n* = 3, *p* < 0.05), # indicates a significant difference compared to the MβCD treatment group with the infected group (*n* = 3, *p* < 0.05).

**Figure 8:**
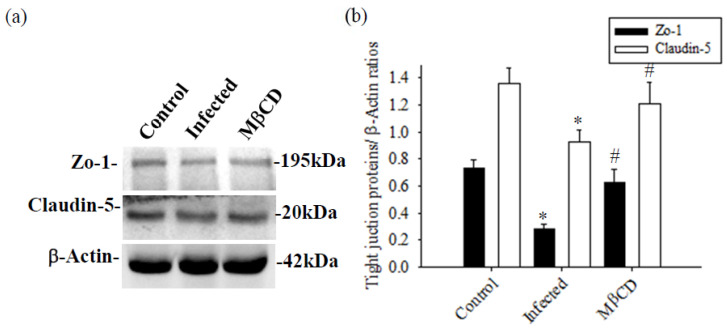
The expression of tight junction proteins was treated with MβCD in mice infected with *A. cantonensis*. (**a**) Compared to the infected group, the expression of the Zo-1 and claudin-5 protein was significantly reduced when treated with MβCD treatment. β-Actin is the loading control. (**b**) The bands quantified by the iBright CL750 imaging system, the expression of the Zo-1 and claudin-5 proteins showed a significant increase with MβCD treatment. Data from three mice in each group are expressed as Mean ± SD, * indicates a significant difference compared to the infected group with the control group (*n* = 3, *p* < 0.05), ^#^ indicates a significant difference compared to the MβCD treatment group with the infected group (*n* = 3, *p* < 0.05).

Mice infected with *A. cantonensis* received treatment with the specific Caveolae/Cav-1 inhibitor MβCD, Cav-1, p-Cav-1, TLR4, and MyD88 expression levels were significantly decreased in MβCD treatment group compared to the infected group (Figure 7, *p* < 0.05). Concurrently, MβCD treatment significantly reduced the activity of MMP-9, an enzyme known to damage the BBB, in both CSF and brain tissue (Figure 7b). The fluorescence intensity of 40 kDa FITC-dextran in CSF, indicative of the permeability of the BBB, showed a significant increase in the infected group and a significant decrease in the MβCD treatment group compared to the infected group (Figure 7c, *p* < 0.05). Similar trends were observed in changes in brain volume, determined by TTC staining, with a significant reduction observed in the infected group following MβCD treatment compared to the infected group (Figure 7d, *p* < 0.05). Subsequently, the levels of Zo-1 and claudin-5, crucial tight junction proteins in the brain, increased significantly in MβCD treatment group compared to the infected group (*p* < 0.05), although still slightly lower than in the control group (Figure 8).

## 4. Discussion

Cerebral edema frequently manifests in response to various triggers, such as infection, stroke, and traumatic brain injury. Multiple factors influence the development of brain edema, including activation of inflammatory pathways, disruption of the blood–brain barrier (BBB), dysregulation of ion channels, and changes in osmotic gradients. Increasing experimental and clinical evidence underscores the link between BBB dysfunction and various severe central nervous system (CNS) disorders, including multiple sclerosis, stroke, brain tumors, epilepsy, and Alzheimer’s disease [23]. In an animal model of traumatic brain injury, an observed increase of approximately 6% in brain water content has been documented [24]. Furthermore, the neuropathological changes resulting from *Toxoplasma gondii* infection lead to localized vascular edema, contributing to an overall increase of approximately 1% in the water content [25]. In *A. cantonensis* that causes eosinophilic meningitis, the group infected with *A. cantonensis*, compared to the control group, exhibited a 12% increase in brain volume and an 8% increase in brain water content. This resulted in cerebral hemorrhage, brain edema, and inflammatory responses involving cellular factors. Changes in cerebrospinal fluid (CSF) composition, including total protein, white blood cells, and eosinophils, were correlated with BBB permeability [2,9,26,27]. Interesting, in the context of acute focal cerebral ischemia, significant areas of brain tissue necrosis appear as white regions in TTC staining [28]. However, when mice are infected with *A. cantonensis*, which causes eosinophilic meningitis, TTC staining to measure brain volume does not reveal significant areas of necrosis of brain tissue. This suggests that although there is substantial infiltration of immune cells in the subarachnoid space and ventricles, brain tissue exhibits demyelination [6]. The extent of brain injury may not reach the necrotic state or areas of damaged brain tissue near the subarachnoid space may be minimal, making it difficult to observe. More research is needed to explore these intriguing findings.

Caveolin-1 is present in various components of the cerebral neurovascular unit, including endothelial cells, astrocytes, and neurons. In particular, Cav-1 is particularly abundant in endothelial cells, where it plays a crucial role in the regulation of BBB permeability by influencing endothelial transcytosis and modulation of tight junction proteins [18,19,20,21]. In conditions such as ischemia and lipopolysaccharide-induced injury, Cav-1 expression increases early, contributing to a decrease in claudin-5 and occludin, resulting in the breakdown of the BBB. Subsequent up-regulation of Cav-1 leads to degradation of tight junction proteins (claudin-5 and occludin), exacerbating damage to the BBB and increasing permeability [16,29]. In hypoxic brain edema, Cav-1 forms membrane invaginations, facilitating claudin-5 endocytosis and subsequent autophagy, ultimately increasing BBB permeability and causing cerebral edema [30]. In ischemic brain injury, tissue-type plasminogen activator (tPA) treatment activates matrix metalloproteinase-9 (MMP-9) directly in vascular endothelial cells or indirectly through Cav-1, promoting MMP-9 activation and subsequent degradation of tight junction proteins. This process leads to the breakdown of the BBB and increased permeability [31,32]. Interestingly, tPA, through Cav-1, also inhibits the activity of MMP-9 to protect the BBB and reduce damage [33]. In a previous study, infection with *A. cantonensis* in mice resulted in increased expression of plasminogen activators and MMP-9, leading to damage to the BBB, degradation of the extracellular matrix (ECM), and infiltration of immune cells, leading to eosinophilic meningitis [9]. To investigate the involvement of Cav-1 in the regulation of BBB permeability, we evaluated MMP activity and expression, as well as BBB permeability. The expression of the Cav-1 protein is positively correlated with brain edema, BBB permeability, and MMP-9 activity in infection-induced meningoencephalitis with *A. cantonensis*. To further elucidate the role of Cav-1 in the regulation of BBB permeability, we employed methyl-β-cyclodextrin (MβCD), a cholesterol depleting agent that disrupts caveolae by inhibiting Cav-1 activity [34]. Treatment with MβCD, which prevents caveola formation, resulted in a Cav-1-induced decrease in meningoencephalitis in *A. cantonensis* infection, leading to a decrease in MMP-9 activity in the brain and CSF, as well as degradation of tight junction proteins (Zo-1 and claudin-5), thus mitigating damage to the BBB. Cav-1/MMP-9 signaling cascades play a critical role in mediating BBB damage that causes cerebral hemorrhage and brain edema during cerebral ischemia-meningoencephalitis induced by *A. cantonensis* infection.

Traumatic brain injury improves pro-inflammatory responses, neuronal loss, and long-term behavioral deficits, for which Cavs can regulate cytokine production to regulate neuronal and glial survival signaling [35]. In Cav-1 knockout mice, there is a reduction in heme oxygenase-1 (HO-1), macrophage inflammatory protein 2, MMP-9, and COX-2 during hemorrhagic stroke, leading to decreased immune cell infiltration and mitigated hemin-induced neuronal toxicity, ultimately improving brain damage [36]. It is interesting that Cav-1 plays a neuroprotective role by modulating cytokine expression and thus mitigating neuroinflammation. Cav-1 signaling through the Cav-1/VEGF pathway promotes both neural regeneration and vascular formation, while simultaneously reducing apoptosis. This multifaceted approach appears to alleviate brain damage caused by oxidative stress [33]. Cav-1, by binding to eNOS, inhibits the activation of eNOS, reducing the production of toxic NO. Furthermore, it suppresses the activation of MMP-9, reducing the degradation of tight junction proteins such as claudin-5 and occludin in the BBB. This attenuation mitigates the disruption of the BBB and the consequent extracellular brain edema. Cav-1 also regulates inflammatory responses in the vascular system by modulating the expression of receptors for inflammatory molecules such as TNF-α, thus reducing the ability of endothelial cells to maintain the integrity of the inner layer [21,37,38,39,40]. Consistent with previous studies, NF-κB and pro-inflammatory factors, including tumor necrosis factor-α (TNF-α), interleukin-1β (IL-1β), IL-5, HO-1, and (peroxisome proliferator activated receptor) PPAR-γ also participate in the regulatory mechanisms governing *A. cantonensis*-induced disruption of the BBB, leakage of immune cells into the subarachnoid space, and subsequent eosinophilic meningitis and brain damage. In *A. cantonensis* infection, the expression of HO-1 and COX-2 in the mouse brain increases, indicating their participation in regulation and their association with MMP-9 [27,41]. Our investigation of the role of Cav-1 in *A. cantonensis*-induced eosinophilic meningoencephalitis, which leads to brain damage, cerebral hemorrhage, and cerebral edema, revealed an increase in Cav-1 levels in the brains of infected mice. This increase was correlated with increased BBB permeability and inflammation, suggesting that Cav-1 may contribute to inflammation and damage to the BBB by increasing the production of pro-inflammatory cytokines.

Cav-1 interacts with Toll-like receptor 4 (TLR4), activating downstream inflammatory signals and exacerbating neuroinflammation and BBB damage [42]. The activation of the TLR4/MyD88 signaling pathway through MAPK, ERK1/2, and JNK leads to the regulation of NF-κB and AP-1, thus facilitating upregulation of pro-inflammatory cytokines. [42,43,44]. In the pathogenesis of neuroinflammatory-related diseases, activated astrocytes under inflammatory conditions, through the TLR4/MyD88 signaling pathway, regulate the expression of pro-inflammatory molecules via NF-κB, MAPK, and Jak1/Stat1 pathways, influencing the inflammatory responses of surrounding cells [45,46]. Furthermore, activation of the TLR4/MyD88 signaling pathway regulates the expression of VCAM-1 and ICAM-1 in endothelial cells, facilitating the passage of immune cells through the BBB, but also leading to breakdown of the BBB. Inhibition of TLR4 can reduce damage to the BBB [47]. In cerebral pathologies such as ischemic brain injury, cerebral hemorrhage, and cerebral edema, increased expression of Cav-1 contributes to increased BBB permeability, exacerbating symptoms of brain injury. After ischemic stroke, cortical neuronal death, and hypoxia-induced astrocyte apoptosis, Cav-1, through the Ras/Ras/ERK signaling pathway, protects against hypoxia-induced astrocyte apoptosis. Furthermore, through the Src kinase and ERK 1/2 signaling pathways, it prevents ischemic cell death in neurons [48,49]. After cerebral ischemia, miR-199a-5p regulates Cav-1 expression, leading to increased expression of BDNF and VEGF, promoting neural stem cell differentiation and endogenous neurogenesis [50]. Therefore, in neuroinflammatory conditions, TLR4 through the MyD88/NF-κB signaling pathway, regulates the expression of pro-inflammatory factors, leading to increased permeability of the BBB and neuronal cell damage. Previous investigations have revealed that the infection-induced increase in MMP-9 induced by *A. cantonensis* is governed by the NF-kB or MAPK/ERK/AP-1 pathways. NF-κB and AP-1, as ubiquitous transcription factors, interact with DNA to regulate the expression of inflammatory genes, thus modulating inflammation [51,52]. Consequently, it is speculated that in *A. cantonensis* infected mice, leading to eosinophilic meningoencephalitis, MβCD can negatively regulate Cav-1, subsequently degrading tight junction proteins of the TLR4/MyD88 signaling pathway, thus altering the permeability of the BBB and ultimately contributing to cerebral hemorrhage and cerebral edema. It is hypothesized that infection with *A. cantonensis* activates the TLR4/MyD88 signaling pathway through Cav-1 phosphorylation, leading to increased activity of the NF-kB or MAPK/ERK/AP-1 signaling pathway, subsequently increasing MMP-9 activity, resulting in damage to the BBB, increased permeability, and inflammatory responses.

The CNS is protected against the fluctuating environment of blood by two crucial barriers: the BBB and the blood-cerebrospinal fluid barrier (BCSFB). The BBB, located at the level of endothelial cells within the CNS microvessels, ensures strict regulation, while the BCSFB is formed by choroid plexus epithelial cells. Despite their distinct locations, both barriers share similar structures and functions. Notably, when assessing permeability using 40 kDa FITC-dextran, it becomes challenging to discern whether leakage originates from the BBB or the BCSFB. Cav-1 plays a key role in modulating BBB permeability by regulating endocytosis of endothelial cells, tight junction proteins, and adherent junction proteins. Through experimentation involving MβCD treatment to inhibit Cav-1 effects on endothelial cells, a significant decrease in 40 kDa FITC-dextran concentration was observed (Figure 7c). This suggests that permeability primarily relates to the BBB, although the influence of the BCSFB cannot be completely disregarded. Further examination of BBB permeability was conducted by an in vivo permeability assay.

Caveolae/caveolins in endothelial cells have emerged as a crucial pathway for the invasion of pathogenic microorganisms into the body. Caveolae, through nonclathrin-regulated macropinocytosis, provide a pathway for the cellular internalization of numerous molecules. Unlike clathrin-mediated endocytosis, caveolae play a distinct role in the entry of various pathogens such as viruses, bacteria, parasites, and toxins into cells. Through the caveolae mechanism, pathogens invading host cells can escape lysosomal fusion and escape host cell lysosomal killing mechanisms [53,54,55]. In the context of HIV infection, PPARα and PPARγ, regulated by Cav-1, modulate the ERK and Akt signaling pathways to reduce the expression of MMP-9, thus mitigating the damage to the BBB [56,57]. In parasitic infections, host macrophages use caveolae to engulf *Leishmania chagasi*. This process delays the fusion of *L. chagasi* with lysosomes, resulting in increased intracellular survival of *L. chagasi* [58]. *Trichomonas vaginalis*, in addition to its interaction with glycosaminoglycans on the surface of the host cell and its specific binding to heparan sulfate on proteoglycans, also enters host cells through caveolae-regulated endocytosis dependent on host cell cholesterol. This process occurs independently of clathrin-mediated endocytosis, using lipid-raft-mediated endocytosis [59]. Unlike the invasion mechanisms of protozoans, during infection by the nematode *A. cantonensis*, an increase in Cav-1 expression in the brain and through the TLR4/MyD88 signaling pathway to activate MMP-9 leads to degradation of tight junction proteins (Zo-1 and claudin-5). This modulation alters the permeability of the BBB, resulting in infiltration of immune cells and brain edema.

## 5. Conclusions

The crucial role of Cav-1 in *A. cantonensis*-induced eosinophilic meningoencephalitis involves regulating the expression of MMP-9 through the TLR4/MyD88 signaling pathway, leading to degradation of the BBB and contributing to cerebral complications, hemorrhage, and edema.

## Data Availability

Data are contained in the article.
